# Hospital admissions after vertical integration of general practices with an acute hospital: a retrospective synthetic matched controlled database study

**DOI:** 10.3399/bjgp20X712613

**Published:** 2020-09-08

**Authors:** Victor Yu, Steven Wyatt, Michael Woodall, Sultan Mahmud, Vijay Klaire, Karla Bailey, Mohammed Amin Mohammed

**Affiliations:** NHS Midlands and Lancashire Commissioning Support Unit, West Bromwich.; NHS Midlands and Lancashire Commissioning Support Unit, West Bromwich.; NHS Midlands and Lancashire Commissioning Support Unit, West Bromwich.; Royal Wolverhampton Hospitals NHS Trust, Wolverhampton.; Royal Wolverhampton Hospitals NHS Trust, Wolverhampton.; Royal Wolverhampton Hospitals NHS Trust, Wolverhampton.; NHS Midlands and Lancashire Commissioning Support Unit, West Bromwich.

**Keywords:** primary health care, retrospective studies, synthetic controls, unplanned hospital admissions, vertical integration

## Abstract

**Background:**

New healthcare models are being explored to enhance care coordination, efficiency, and outcomes. Evidence is scarce regarding the impact of vertical integration of primary and secondary care on emergency department (ED) attendances, unplanned hospital admissions, and readmissions.

**Aim:**

To examine the impact of vertical integration of an NHS provider hospital and 10 general practices on unplanned hospital care

**Design and setting:**

A retrospective database study using synthetic controls of an NHS hospital in Wolverhampton integrated with 10 general practices, providing primary medical services for 67 402 registered patients.

**Method:**

For each vertical integration GP practice, a synthetic counterpart was constructed. The difference in rate of ED attendances, unplanned hospital admissions, and unplanned hospital readmissions was compared, and pooled across vertical integration practices versus synthetic control practices pre-intervention versus post-intervention.

**Results:**

Across the 10 practices, pooled rates of ED attendances did not change significantly after vertical integration. However, there were statistically significant reductions in the rates of unplanned hospital admissions (−0.11, 95% CI = −0.18 to −0.045, *P* = 0.0012) and unplanned hospital readmissions (−0.021, 95% CI = −0.037 to −0.0049, *P* = 0.012), per 100 patients per month. These effect sizes represent 888 avoided unplanned hospital admissions and 168 readmissions for a population of 67 402 patients per annum. Utilising NHS reference costs, the estimated savings from the reductions in unplanned care are ∼£1.7 million.

**Conclusion:**

Vertical integration was associated with a reduction in the rate of unplanned hospital admissions and readmissions in this study. Further work is required to understand the mechanisms involved in this complex intervention, to assess the generalisability of these findings, and to determine the impact on patient satisfaction, health outcomes, and GP workload.

## INTRODUCTION

Healthcare systems are facing multiple challenges, which include increasing demand and resource constraints. It is argued that one way to address some of these challenges is to provide greater integration between healthcare providers.^1^^,^^2^ Horizontal integration is defined as when two or more organisations or services delivering care at a similar level come together. Examples include mergers of acute hospitals and the formation of organisations such as care trusts that bring together health and social care.^3^^,^^4^ In comparison, vertical integration occurs when two or more organisations or services providing care at different levels are joined into a single organisational structure. Examples include mergers of acute hospitals and community health services, and tertiary care providers working with secondary care providers.^5^^,^^6^

Although some examples of vertical integration have been reported,^7^^,^^8^ there is a paucity of evidence evaluating the impact of vertical integration. This article examines the impact of a vertical integration project involving 10 general practices and one acute NHS hospital and community service provider in the West Midlands, which took over staffing, contracts, finance, and payroll functions, allowing GPs to concentrate on supporting patients using a shared management information system and live patient data to track patients as they move from primary to community and hospital services. A key objective of this vertical integration project was to reduce emergency hospital use arising from the poor management of patients’ long-term conditions. It was posited that this could be achieved by increasing patients’ access to primary care and increasing GPs’ access to secondary care consultants.

The aim of this study was to assess the impact of vertical integration on unplanned (emergency) care using retrospective synthetic controls (SC) methodology to evaluate this complex intervention. Synthetic control methods have become increasingly popular for estimating the causal effects of policy interventions in a variety of applications,^9^^,^^10^ including examining the effect of the Quality and Outcomes Framework (QOF) national primary care pay-for-performance programme on population mortality.^11^

## METHOD

### Study design

A synthetic controls retrospective database study design was used to evaluate the causal effect of vertical integration on three health outcome variables of interest: emergency department (ED) attendance rates, unplanned hospital admission rates, and unplanned hospital readmission rates. This was achieved by comparing these rates in the 10 vertical integration practices versus SC practices, before and after the intervention.

**Table:** How this fits in

There is widespread interest in integrating different forms of healthcare provision to improve patient outcomes. Vertical integration is defined as when two or more organisations or providers of care at different levels are integrated into a single organisational structure. Few studies have explored the impact of integrating primary and secondary care on healthcare use. This quasi-experimental study explored the impact of integrating 10 GP practices with an acute hospital and found a modest but statistically significant reduction in unplanned hospital admissions and readmissions.

### Setting and study population

The Royal Wolverhampton NHS Trust (RWT) is a large acute hospital and community service provider situated in the metropolitan borough of Wolverhampton in the West Midlands county of England. Serving a population of 260 000 people, the trust has approximately 900 beds and 9400 staff. In June 2016, RWT began a vertical integration programme by assuming responsibility for the provision of primary care services and, by February 2018, it was responsible for the operations and delivery of 10 of the 42 GP practices in Wolverhampton. (These GP practices were initially engaged with key management figures in the trust over discussions of issues that led to the development of the vertical integration programme. In addition, the practices actively chose to be part of the vertical integration project.)

These 10 vertical integration practices in Wolverhampton are responsible for the care for 67 402 patients. One GP practice left the vertical integration project in July 2018 but is still included in the analyses up to that point. All practices have been de-identified in this analysis and the reason(s) for one GP practice leaving are subject to a nondisclosure agreement.

A donor pool of general practices was identified from Wolverhampton and nearby areas. Based on geographical proximity, a proportion of these practices (41/408), namely those also in the Wolverhampton Clinical Commissioning Group (*n* = 32) and South East Staffordshire and Seisdon Peninsula Clinical Commissioning Group (*n* = 9), would still mainly refer their patients to the RWT acute hospital. For each vertical integration practice, the donor pool was constrained to those with a similar (±20%) practice list size.

### Data

A bespoke dataset was assembled for the purposes of this analysis, which consisted of a range of predictor variables and the three outcomes of interest, for the 10 vertical integration practices and 408 potential donor practices, at monthly intervals between April 2015 and March 2019 (*n* = 48). GP practice was used as the identifier field for data linkage.

Data for the three outcome variables — ED attendances, unplanned hospital admissions, and unplanned hospital readmissions — as well as emergency length of stay (days), were derived from Secondary Uses Service data extracts. These were converted into rates per 100 patients per month.

Population and demographic data were collected from GP register data. Data on ethnicity were unavailable, so, as a proxy, the proportion of registered patients who have been admitted to hospital, who are from black and minority ethnic (BME) groups, was calculated using the ethnicity data recorded in hospital datasets. Measures of disease prevalence were derived from QOF data,^12^ and used to represent markers of control for long-term conditions. Staff count data were extracted from NHS Workforce Data. Index of Multiple Deprivation (IMD) 2015 values were collected from Public Health England’s Fingertips data platform.^13^ GP practice IMD data were calculated using a population-weighted average at lower layer super output area level. ED attendance activity data were used as a proxy to calculate the percentage of patients of a practice designated either as rural or urban, based on the Office for National Statistics’ 2011 rural–urban classification.^14^

### Statistical analyses

All analyses were performed using R (Version 3.6) (https://www.r-project.org/). In addition, Google High Performance Computing was used to optimise run times of the models.

### Synthetic controls

Statistical analyses were carried out using the R package multivariate synthetic control methods using time series (MSCMT),^15^ which offers improved optimisation procedures and computational performance over earlier packages, and facilitates analysis for multiple outcome variables.^16^ Default settings for the optimisation procedure were selected. The SC practices, a linear combination of a set of practices drawn from a pool of ‘donor practices’, were created by matching the vertical integration practice in terms of staffing levels, practice population characteristics, and outcome variables during the pre-intervention period. The MSCMT package also provides estimation of causal effects.

After the construction of the synthetic practices, statistical inference is based on ‘placebo tests’, whereby the analysis is performed as if other units in the donor pool were the treated unit. This process generates a distribution of effect estimates from which standard errors and confidence intervals can be derived, and pooled effect sizes estimated. Statistical significance was defined as *P*<0.05. The effect sizes reported represent the average monthly difference between the pre-intervention value and the average post-intervention value of the vertical integration practice and its SC practice in a difference of difference analysis, per 100 practice population.

The primary aim of the study was to evaluate the vertical integration project as a whole-system transformation, rather than each vertical integration practice individually. The pooled treatment effect was estimated by combining the individual practice effect sizes and standard errors using the inverse variance method (see Supplementary Appendix S1 for details). Higgins’ and Thompson’s *I*^2^ statistic^17^ was used to assess the level of heterogeneity between the individual practice effect sizes, and to decide on whether to select a fixed or random-effects approach for the pooling process.^18^

### Quantifying potential cost savings

Potential cost savings were estimated using national reference costs of each outcome variable from NHS Improvement.^19^ For inpatient spells, the unit cost was estimated by multiplying the reference cost for, say, an unplanned inpatient episode by the ratio of total finished consultant episodes (FCE) to finished admission episodes (FAE) in the 2017/2018 financial year (∼20 million FCE/∼16.6 million FAE)^20^ (see Supplementary Appendix S2 for details). Because it could not be verified whether the avoided unplanned hospital readmissions were a subset of the avoided unplanned hospital admissions, these figures were not calculated to avoid the possibility of double counting.

## RESULTS

### Baseline characteristics of vertical integration practices and synthetic control practices

The vertical integration process commenced in June 2016 through to July 2018 with 10 diverse GP practices whose pre-intervention characteristics are shown in Tables 1 and 2 (see Supplementary Table S2 for details of additional independent covariates).

**Table 1.** Details of study practices in month before joining the vertical integration programme

**General practice number**	**VI join date**	**Number of general practices in donor pool**	**General practice list size**	**Number of GPs**	**Number of nurses**	**BME, %**	**Practice list female 0–19 years, %**	**Practice list female 20–64 years, %**	**Practice list female ≥65 years, %**	**Practice list male 0–19 years, %**	**Practice list male 20–64 years, %**	**Practice list male ≥65 years, %**
Practice #1	June 2016	80	8281	6	4	10.81	10	27	14	10	27	11
Practice #2	June 2016	93 (94)^a^	6644	8	4	53.97	12	30	8	13	31	7
Practice #3^b^	June 2016	83	7674	2	2	17.67	13	28	7	14	31	7
Practice #4	January 2017	84	3467	3	1	33.46	10	29	10	10	34	9
Practice #5	April 2017	92	4356	3	1	13.86	10	29	14	12	25	10
Practice #6	July 2017	92	4377	6	2	31.33	15	30	6	15	30	5
Practice #7	September 2017	75	10 178	7	3	45.80	10	32	4	11	40	3
Practice #8	November 2017	63	11 503	4	5	21.35	10	28	12	11	28	10
Practice #9	February 2018	97	5782	3	2	6.21	9	32	9	11	30	9
Practice #10	July 2018	100	5140	5	1	28.7	11	28	11	11	29	10

a One practice in the donor pool was excluded because of computational errors.

b Practice left the programme in June 2018. BME = black and minority ethnic. VI = vertical integration.

**Table 2.** Average pre-intervention rates for the three outcome variables of the vertical integration practices and their synthetic controls, per 100 population from April 2014 to the month before joining the intervention

**Practice number**	**ED attendance rate VI practices *n* (SD)**	**ED attendance rate SC practices *n* (SD)**	**Unplanned admissions rate VI practices *n* (SD)**	**Unplanned admissions rate SC practices *n* (SD)**	**Unplanned readmissions rate VI practices *n* (SD)**	**Unplanned readmissions rate SC practices *n* (SD)**
Practice #1	2.94 (0.28)	2.89 (0.22)	0.92 (0.16)	0.94 (0.06)	0.13 (0.05)	0.14 (0.02)
Practice #2	2.69 (0.31)	2.69 (0.19)	0.87 (0.13)	0.86 (0.06)	0.12 (0.05)	0.12 (0.02)
Practice #3	3.54 (0.84)	3.51 (0.49)	1.1 (0.34)	1.12 (0.2)	0.15 (0.08)	0.15 (0.04)
Practice #4	2.53 (0.41)	2.5 (0.19)	0.81 (0.15)	0.83 (0.06)	0.13 (0.07)	0.11 (0.02)
Practice #5	2.10 (0.25)	2.13 (0.16)	0.81 (0.15)	0.76 (0.06)	0.12 (0.06)	0.12 (0.02)
Practice #6	3.93 (0.54)	3.76 (0.30)	1.27 (0.29)	1.2 (0.15)	0.2 (0.10)	0.18 (0.04)
Practice #7	2.53 (0.28)	2.47 (0.12)	0.63 (0.09)	0.62 (0.05)	0.07 (0.03)	0.08 (0.02)
Practice #8	2.13 (0.25)	2.12 (0.14)	0.71 (0.10)	0.69 (0.05)	0.08 (0.02)	0.09 (0.01)
Practice #9	1.84 (0.25)	1.91 (0.15)	0.54 (0.10)	0.6 (0.07)	0.06 (0.03)	0.07 (0.02)
Practice #10	2.79 (0.41)	2.78 (0.20)	1.02 (0.21)	1.03 (0.08)	0.17 (0.07)	0.17 (0.03)

ED = emergency department. SC = synthetic control. VI = vertical integration.

**Figure 1.**
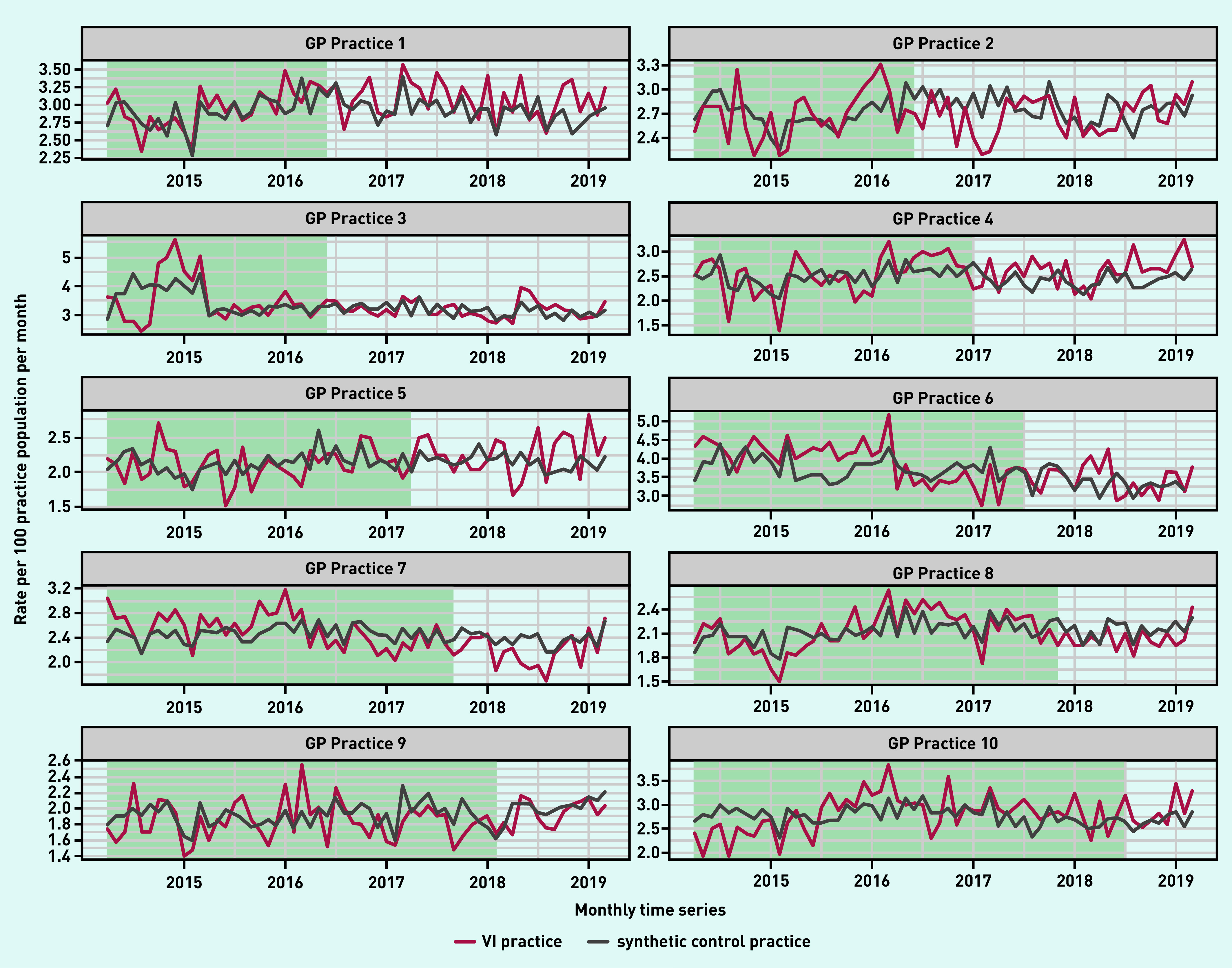
***Emergency department attendance rates of vertical integration practices with their synthetic control. Pre-intervention period is shaded.*** ***VI = vertical integration.***

**Figure 2.**
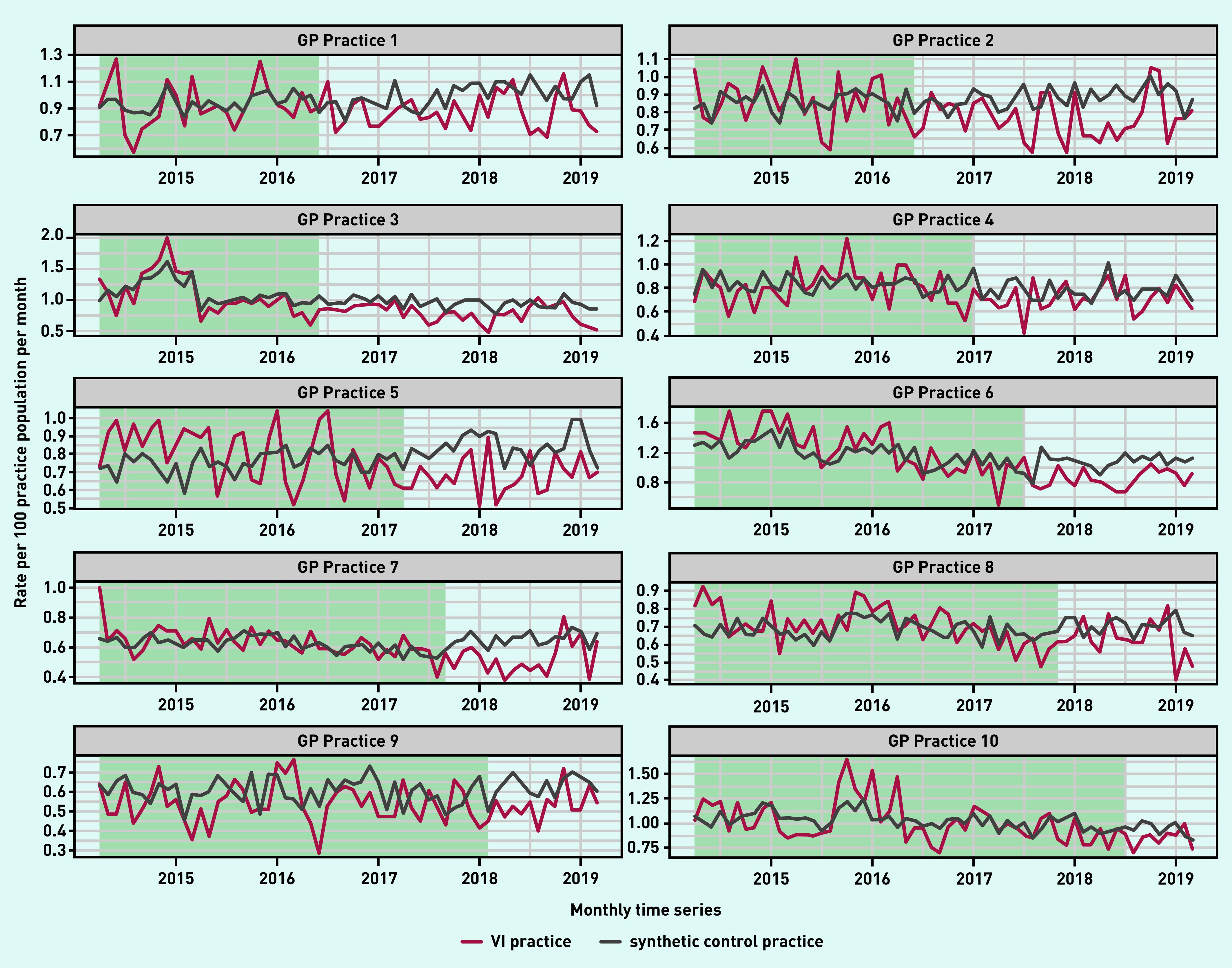
***Unplanned hospital admission rates of vertical integration practices with their synthetic control. Pre-intervention period is shaded.*** ***VI = vertical integration.***

**Figure 3.**
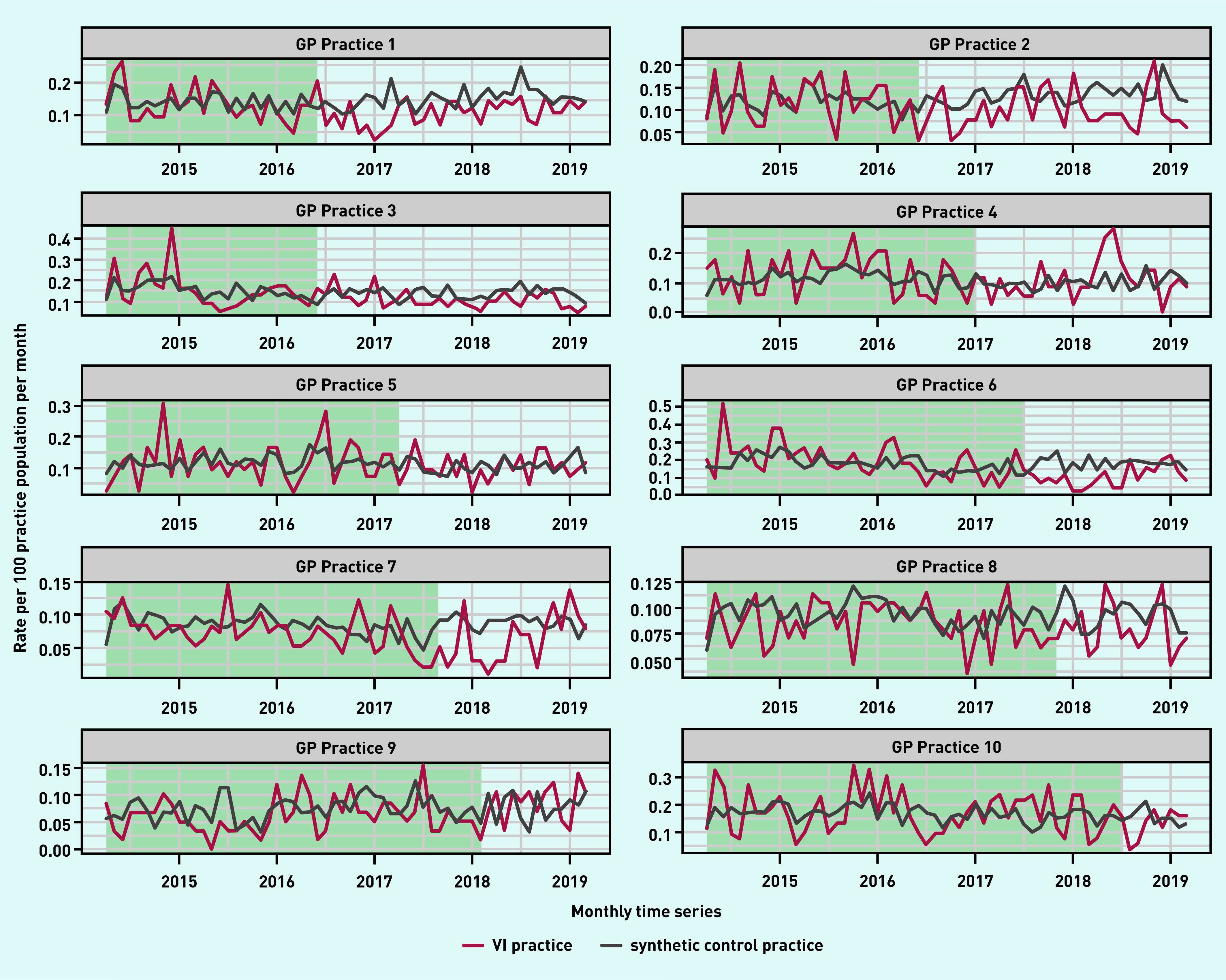
***Unplanned hospital readmission rates of vertical integration practices with their synthetic control. Pre-intervention period is shaded.*** ***VI = vertical integration.***

As Table 1 shows, the list sizes of the 10 vertical integration practices ranged from 3467 to 11 503, the number of GPs ranged from two to eight, and the number of nurses ranged from one to five. The percentage of BME patients ranged from 6.21% to 53.97%, the percentage of male patients aged ≥65 years ranged from 3% to 11%, and the percentage of female patients aged ≥65 years ranged from 4% to 14%. The majority of the patient population in the vertical integration practices were classified as urban, with QOF profiles that varied across the practices (see Supplementary Table S2 for details). Table 2 shows that the pre-intervention rates for ED attendances, unplanned hospital admissions, and unplanned hospital readmissions for the vertical integration practices and SC practices are not dissimilar. Monthly time-series plots are given in Figures 1 to 3 for each outcome variable in each vertical integration practice and its SC.

Weightings of the synthetic counterparts of the vertical integration practices can be found in Supplementary Table S1.

### Estimated effect sizes

**Figure 4.**
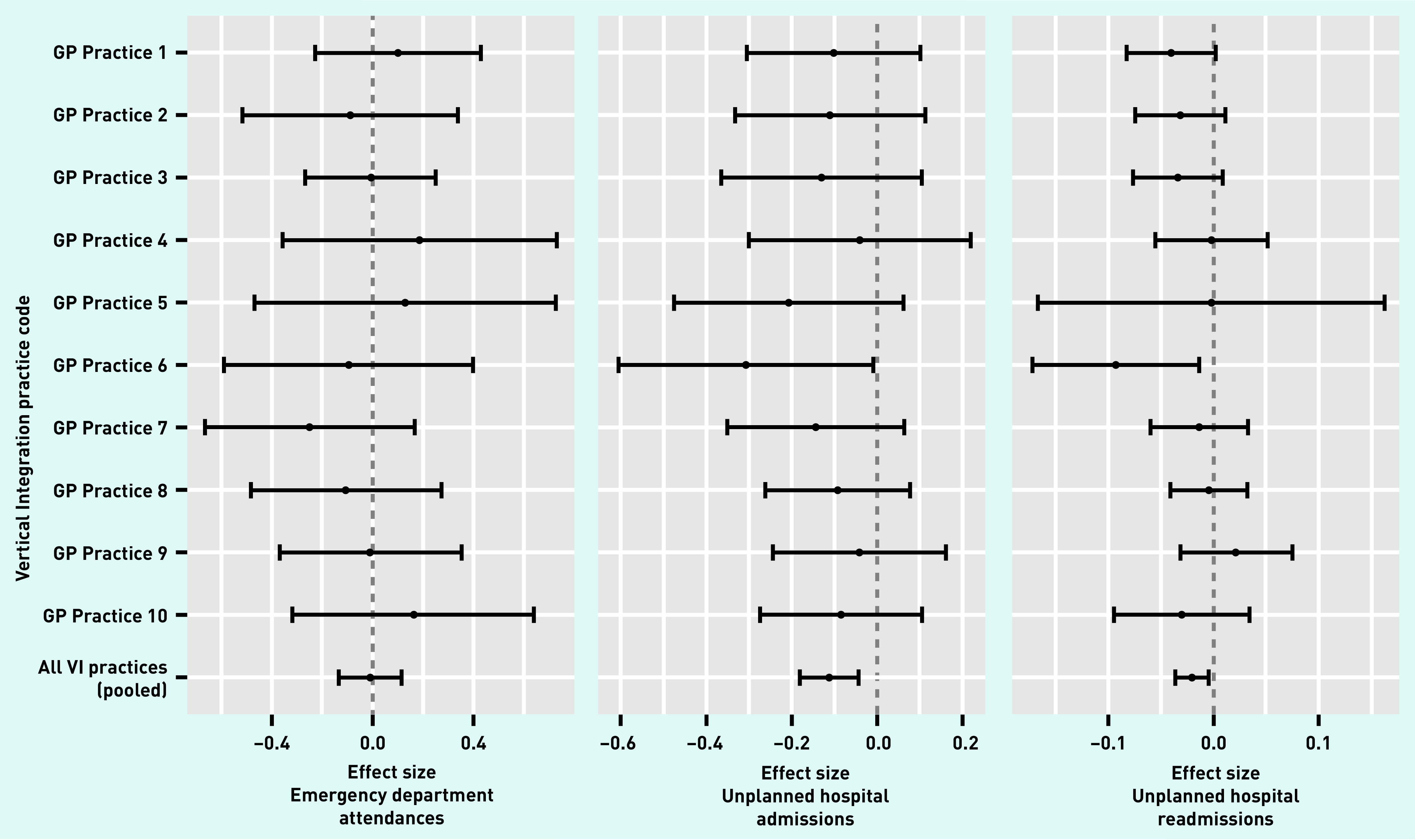
***Effect sizes comparing vertical integration practices with synthetic controls for emergency department attendances (left), unplanned hospital admissions (middle), and unplanned hospital readmissions (right), with 95% confidence intervals, showing changes in rate per month per 100 registered patients (see Supplementary Table S3 for details).***

The effect sizes shown in Figure 4 (see Supplementary Table S3 for numerical results) represent the average monthly difference between the pre-intervention value and the average post-intervention value of the vertical integration practice and its SC practice using a fixed-effects approach, as the test for heterogeneity was not significant (ED *I*^2^: 0.0%, 95% CI = 0.0% to 0.2%, degrees of freedom [df] = 9, *P* = 0.95; unplanned admissions *I*^2^: 0.0%, 95% CI = 0.0% to 0.0%, df = 9, *P* = 0.96; unplanned readmissions *I*^2^: 0.0%, 95% CI = 0.0% to 60.5%, df = 9, *P* = 0.48).

The pooled results show a statistically significant reduction in unplanned hospital admissions (−0.11, 95% CI = −0.18 to −0.045, *P* = 0.0012) and readmissions (−0.021, 95% CI = −0.037 to −0.0049, *P* = 0.012), per 100 patients per month, but not ED attendances.

Based on registered population sizes for the vertical integration practices (67 402 people), these effect sizes equate to a reduction of 74 unplanned admissions and 14 unplanned readmissions a month, which equate to a reduction of 888 unplanned admissions and 168 unplanned readmissions across the vertical integration practices per year. NHS reference costs indicate that the average unit cost for an unplanned inpatient per finished consultant episode is £1603. Multiplying this by the ratio of finished consultant episodes to finished admission episodes in 2017/2018 gives an average unit cost for an unplanned, emergency admission of £1931. This implies a reduction in costs of £1 714 728 (= 888 × £1931) per annum as a result of reductions in unplanned admissions. Sensitivity analysis using lower and upper 95% boundary estimates gives figures of £2 803 812 and £695 160, respectively (Supplementary Appendix 2).

## DISCUSSION

### Summary

This study examined the impact of a complex intervention — vertical integration, involving an acute hospital and 10 GP practices — on unplanned care. Across the 10 practices involved, pooled rates of ED attendances did not change significantly after vertical integration. However, there were significant reductions in the rates of unplanned hospital admissions (−0.11, 95% CI = −0.18 to −0.045, *P* = 0.0012) and unplanned hospital readmissions (−0.021, 95% CI = −0.037 to −0.0049, *P* = 0.012), per 100 patients per month. These effect sizes represent 888 avoided unplanned hospital admissions and 168 readmissions for a population of 67 402 patients per annum. The estimated savings from the reductions in unplanned care are in excess of £1.5 million.

Given the general lack of progress in reducing the demand for unplanned care (a few notable exceptions aside),^21^ these findings suggest that vertical integration appears to remove barriers to more effective coordinated patient care in a diverse set of GP practices, although the detailed mechanisms remain unclear.

### Strengths and limitations

This is an observational study and, as such, does not provide proof of causation. Nonetheless, sophisticated statistical methods were used to estimate a ‘causal’ effect. The background of this study provided appropriate context for the implementation of the SC study design. The nature of the SC design also meant that the explicit control of confounders was unnecessary because synthetic controls incorporate both observed and unobserved time-varying confounders.^22^

The use of an SC study assumed that no other major events differentially impacted potential control practices, or that the intervention did not surreptitiously impact on geographically adjacent, non-treatment practices. Other (non-vertical integration) practices in Wolverhampton were included in the donor pool in an attempt to mitigate this. Nonetheless, it is possible that the observed effect was due in part to a set of wider changes in the Wolverhampton area along with the vertical integration programme (see Supplementary Figure S1 for details). It is also possible that the findings are compromised by not fully capturing the unplanned activity of practices in the donor pool that were outside the catchment area of the target hospital, as this might have led to unaccounted differences in the SC outcome estimates, owing to varying care quality.

The significant size of the study population across multiple diverse GP practices and their synthetic controls strengthens the reliability of the findings and reduces the risk of bias through exclusion of subgroups. Nonetheless, the study was based in one health economy and further work would be required to assess the generalisability of the findings.

Although the causal mechanisms involved were not examined, there are several potential explanations for the findings.

RWT sought to increase the accessibility of primary care and made appointments available for patients to book. This has been delivered, in part, by adjusting the skill mix in practices, and ensuring that appointments are handled by the most appropriate staff. If this increase in appointments has occurred at a greater rate than control practices, and these additional appointments have been taken up by patients, and these appointments have been used to improve the control of patients’ long-term conditions, then this may have led to a reduction in GP-referred unplanned hospital admissions. Given that no decreases in the rate of ED attendances were observed, it seems unlikely that these additional appointments have been used to manage patients’ urgent care needs.

RWT has developed a management information system for GP practices joining the vertical integration scheme, which provides data on patients’ use of primary, community, and hospital services. If this information system provides superior insight and access to GPs, then it may enhance GPs’ surveillance of their at-risk population and support the creation of novel interventions to reduce their risk of admission.

As a result of the vertical integration programme, GPs are represented on a number of RWT’s management committees. This, along with the process of employing practice staff directly, may increase the extent of joint working and coordination between primary and secondary care. If GPs can access the advice of secondary care clinicians more readily, this might lead to a greater confidence in managing patients without the need for admission through sharing the clinical risks between primary and secondary care, which may lead to a reduction in unplanned admissions.

Finally, RWT has committed to the principles of population health management and now employs several public health specialists. This increased emphasis on systems thinking may reduce the reliance on hospital care.

### Comparison with existing literature

Unplanned hospital admissions in the UK rose by 47% between 1998 and 2013, from 3.6 million to 5.3 million, with only a 10% increase in population over this period. These admissions are expensive: in 2012 they cost the NHS £12.5 billion.^23^

Although reviews suggest that risk prediction models may have a role to play in identifying those people who are at risk of unplanned admission to hospital, their impact on unplanned care remains limited, with suggestions that they may increase unplanned care.^24^^,^^25^ The authors of the PRISMATIC study suggest that risk stratification tools may increase emergency hospital admissions by focusing GPs’ attention on patients at the highest risk of admission at the expense of patients with lower but more modifiable risks.^26^ More promising approaches based on greater integration between primary care and community services have been advocated, with preliminary evidence of a 14% reduction in unplanned emergency admissions against a 28.5% increase in the background. (The 14% reduction represents the change in Frome, a small town in Somerset — where the integration described by Abel *et al* happened — and the 28.5% increase represents a change in the rest of Somerset, excluding Frome.)^21^

The study findings with respect to unplanned hospital readmissions corresponds with the findings of a vertical integration study in Portugal, which also found that vertical integration has the potential to reduce hospital readmissions.^27^ However, a study of vertical integration in the US noted that the process of organisational change led to increases in emergency hospital use, with limited evidence of enhanced quality of care.^28^

### Implications for research

This study contributes to the evidence base on the validity of vertical integration as a means of improving service coordination and reducing unplanned hospital use, although many questions remain unanswered and require further study. Although teasing out cause and effect relationships in a complex adaptive system is challenging, perhaps the most crucial question is to determine how and why vertical integration has been effective. The mechanisms that led to lower unplanned care need to be studied. The wider intended or unintended consequences of vertical integration also need attention, as do the costs of vertical integration. Moreover, further studies are required to assess the generalisability of the findings.

Notwithstanding the limitations, the current study offers some evidence that vertical integration may be useful in addressing the year-on-year increase in unplanned hospital admissions in the UK.
